# Effects of preoperative low-intensity training with slow movement on early quadriceps weakness after total knee arthroplasty in patients with knee osteoarthritis: a retrospective propensity score-matched study

**DOI:** 10.1186/s13102-020-00223-7

**Published:** 2020-11-27

**Authors:** Yusuke Kubo, Shuhei Sugiyama, Rie Takachu, Takeshi Sugiura, Masahiro Sawada, Kaori Kobori, Makoto Kobori

**Affiliations:** Department of Rehabilitation, Kobori Orthopedic Clinic, 548-2 Nearaichou, Kita-ku, Hamamatsu City, Shizuoka, 433-8108 Japan

**Keywords:** Exercise preconditioning, Ischemic preconditioning, Ischemia-reperfusion injury, Knee swelling, Low-intensity training, Prehabilitation, Quadriceps weakness, Slow movement, Thigh swelling, Total knee arthroplasty

## Abstract

**Background:**

Severe and early quadriceps weakness (QW) after total knee arthroplasty (TKA), which is caused by acute inflammation resulting from surgical trauma and tourniquet-induced ischemia-reperfusion (IR) injury, can be especially problematic. We focused on tourniquet-induced IR injury, because it has been shown to be preventable through ischemic and exercise preconditioning. Low-intensity resistance exercise with slow movement and tonic force generation (LST) share some similarities with ischemic and exercise preconditioning. The present study primarily aimed to clarify the efficacy of preoperative LST program as prehabilitation for early QW among patients with TKA using propensity score matching analysis.

**Methods:**

This single-center retrospective observational study used data from patients with knee osteoarthritis (*n* = 277) who were scheduled to undergo unilateral TKA between August 2015 and January 2017. Those with missing outcome data due to their inability to perform tests were excluded. The LST group included participants who performed LST and aerobic exercise (LST session) more than seven times for three months prior to surgery. The control group included participants who performed less than eight LST sessions, a general and light exercise or had no exercise for three months prior to surgery. Knee circumference, thigh volume, knee pain during quadriceps strength test (QST) and timed up and go test (TUG), quadriceps strength, and TUG were measured before and 4 days after surgery. Knee swelling, thigh swelling, Δknee pain, QW, and ΔTUG were determined by comparing pre- and postoperative measurements.

**Results:**

Propensity score matching generated 41 matched pairs who had nearly balanced characteristics. The LST group had a significantly lower knee and thigh swelling, QW, and ΔTUG compared to the control group (all, *p* < 0.05). No significant differences in Δknee pain during the QST and TUG were observed between both groups (both, *p* > 0.05).

**Conclusions:**

The present study demonstrated the beneficial effects of preoperative LST program on knee swelling, thigh swelling, QW, and walking disability immediately after TKA.

## Background

Patients who have undergone total knee arthroplasty (TKA) can be characterized as having significant quadriceps muscle weakness (QW) during the early perioperative phase, which can persist for more than a year [1, 2]. QW has been associated with decreased walking speed and endurance, lowered stair negotiation ability, and increased risk for falls [3–5]. Moreover, QW can cause compensatory strategies during gait, chair rise, and stair climbing, which can then lead to excessive mechanical stress to the contralateral lower extremity joints and the lumbar spine [6–9]. Severe QW in the early phase following TKA can be especially problematic, as it contributes to persistent QW throughout the postoperative course, resulting in long-term functional deficits and chronic overloading of other joints [3–9]. As such, early QW following TKA, the treatment of which can be significantly challenging, needs to be addressed to optimize postoperative recovery.

Several studies have shown that early QW was associated with knee swelling, knee pain, and quadriceps muscle atrophy after TKA [1, 10, 11]. These factors can be attributed to surgical trauma and tourniquet-induced ischemia-reperfusion (IR) injury [12–14], which generates vigorous oxidative stress and inflammatory responses characterized by the toxic reactive oxygen species formation, inflammatory cell recruitment, and endothelial barrier failure, resulting in impaired organ function [15]. Additionally, ischemic and exercise preconditioning (IPC and EPC) have shown to prevent IR injury and are well-known interventions for inhibiting IR injury-induced tissue damage in various organs, including skeletal muscle [16–19]. Therefore, tourniquet-induced early QW can be prevented by IPC and EPC.

IPC and EPC refer to the induction of brief cyclic IR episodes (hypoxic stimulation) and exercise training (exercise stimulation), respectively, which induce endogenous protective mechanisms that confer significant tolerance to subsequent lethal IR injury [20, 21]. Accordingly, the present study focused on a form of exercise called low-intensity resistance exercise with slow movement and tonic force generation (LST) characterized by slow movement with sustained muscle contraction (continuous electromyographic working muscle activity) during exercise [22, 23]. LST has been shown to promote significantly lower peripheral muscle oxygenation during exercise compared to the same intensity exercise performed at normal speeds (LST method: 3 s each of eccentric and concentric actions and a 1-s pause with no rest between each repetition; normal speed method: 1 s each of concentric and eccentric actions and a 1-s rest between each repetition) [22]. In other words, LST is a form of exercise that confers both hypoxic and exercise stimulation to the skeletal muscles, thereby providing additive IPC and EPC effects on IR-induced skeletal muscle damage.

## Methods

### Aim, study design, participants, and setting

The present study primarily aimed to clarify the efficacy of preoperative LST program as prehabilitation for early QW among patients with TKA using propensity score matching analysis. We hypothesized that the LST group would have lower knee and thigh swelling and knee pain, resulting in lower QW and walking disability immediately after TKA, compared to the non-LST group (control group).

This single-center retrospective observational study evaluated the efficacy of preoperative LST program as prehabilitation for early QW after TKA at an orthopedic clinic in Japan. This study used data from participants who met the inclusion and exclusion criteria. The inclusion criteria were patients with knee osteoarthritis (Kellgren-Lawrence grade 3 or 4) who were scheduled to undergo unilateral TKA between August 2015 and January 2017. The exclusion criteria were those with missing outcome data (e.g., muscle strength and walking ability) due to their inability to perform tests. Participants were required to visit the orthopedic clinic three months and one month before surgery to undergo preoperative examination for TKA. Only those who were able to visit the orthopedic clinic regularly until surgery were prescribed prehabilitation once a week from three months before surgery. Outcome measurements among participants who underwent prehabilitation and those who did not undergo prehabilitation were evaluated approximately one week before surgery and approximately one month before surgery, respectively, and again on postoperative day 4. Data were collected by four physiotherapists with extensive training in performing measurements. Perioperative care was similar to that described in a previous study [24].

### Prehabilitation

Individual prehabilitation was provided to participants who were divided into two treatment categories—by five physiotherapists at an orthopedic clinic. Participants in category 1 were those who could complete LST with light or moderate pain during exercise. Category 1 included LST and aerobic exercise with a cycle ergometer (heart rate < 120 beats per minute, 15–20 min). The LST program was based on some earlier programs [22, 23]. The types of resistance exercise in LST were squats, forward lunges, and bilateral knee extensions in a seated position. Squats and forward lunges were performed using bodyweight as the load. Bilateral knee extensions were performed at 30% of maximum isometric voluntary contraction with a bilateral isotonic knee extension machine (WT-L02; Minato Medical Science Co., Ltd., Osaka, Japan). Maximum isometric voluntary contraction was measured using the same bilateral knee extension machine at a knee angle of 90° (0° = full extension). The three types of LST resistance exercises were performed as 3 sets of 10 repetitions with slow movement and tonic force generation (3-s eccentric, 5-s isometric, and 3-s concentric actions without rest between repetitions). The rest period between resistance exercise items and between sets was 60 s.

Participants in category 2 were those unable to perform LST due to severe pain during exercise. Category 2 included general and light exercises, such as low-intensity knee extensions and knee range of motion exercises on a therapy couch bed (20 min). Participants in both categories received thermotherapy (hot pack, 10 min) and electrotherapy (Interferential Current Equipment, 10 min).

### Outcome measurement

Pre- and postoperative evaluation comprised measurements of knee circumference at 1 cm and 10 cm proximal to the upper edge of the patella, knee pain during the quadriceps strength test (QST) as well as timed up and go test (TUG), quadriceps strength, and the TUG. Thigh volume was calculated using the truncated cone method through the following formula: V = 1/3πh (a2 + ab + b2), where V represents thigh volume, h represents the distance between two points in the knee circumference (i.e., 9 cm), a represents knee circumference (1 cm), and b represents knee circumference (10 cm). This method has shown excellent criterion-related validity and intra-rater reliability, as well as good inter-rater reliability [25]. Stair climb test (SCT) and Japanese Knee Osteoarthritis Measure (JKOM) scores [26], an index of disease-specific and patient-derived quality of life, were measured only before surgery. Relative changes (%) in knee circumference (1 cm and 10 cm), thigh volume, quadriceps strength, and the TUG were calculated using the formula [(postoperative value − preoperative value) / preoperative value × 100] to determine knee swelling (1 cm and 10 cm), thigh swelling, QW, and ΔTUG, respectively. Given that knee pain involved 0, the absolute change was calculated using the formula (postoperative value − preoperative value) to determine Δknee pain. Preoperative characteristics of the participants and tourniquet time were obtained from their medical records. The primary outcome was QW. Secondary outcomes were knee and thigh swelling, Δknee pain, and ΔTUG.

### Knee circumference

Knee circumference among participants relaxed in the supine position with knees extended was measured at 1 and 10 cm proximal to the upper edge of the patella using a non-stretchable tape measure. Two measurements were performed with mean of the two recordings being used for analysis. Circumference measurements using a tape measure have been shown to have excellent intra-rater reliability and good inter-rater reliability [27, 28].

### Quadriceps strength test

Quadriceps strength was measured using the maximum voluntary isometric contraction via a pull-type hand-held dynamometer (Mobie; Sakai Medical Co., Ltd., Tokyo, Japan) as previously described [24]. Participants were tested in a seated position with a hip angle of approximately 90° and a knee angle of 75° (0° = full extension) while gripping both sides of the couch. Each participant performed two warm-up trials followed by three maximal contractions with a 1-min rest interval. The highest measurement of two valid trials was used for analysis. Subsequently, quadriceps strength was expressed as the maximum voluntary torque per kg body mass using the external lever arm length and body mass of each participant (Nm/kg). Similar quadriceps strength measurements have been reported to have excellent intra-rater reliability and good inter-rater reliability [29].

### Performance test (timed up and go test and stair climb test)

The TUG required participants to rise from a chair (height: 45 cm), walk 3 m, turn around, walk back to the chair, and sit down. Meanwhile, the SCT required participants to ascend and descend a set of 12 steps (high: 18 cm). Participants were allowed to use a T-handle cane and/or a single handrail during both tests if necessary. Both tests were timed twice using a stopwatch, with their mean of the two recordings being used for analysis. Both tests have been found to have excellent reliability [30].

### Visual analog scale test

Pain in and around the knee immediately after the QST and TUG were evaluated using the visual analog scale (VAS), which is presented as a 100-mm line anchored by verbal descriptors (usually ‘no pain’ and ‘worst imaginable pain’). Each participant was asked to make a mark on the 100-mm line that would indicate pain intensity. Participants’ scores were determined by measuring the distance from the zero anchor (“no pain”) to the participant’s mark. The strongest pain intensity during the QST and TUG was used for analysis. The VAS test has been reported to have excellent reliability [31].

### Sample size

Sample size calculation was performed with QW as the primary outcome. Because a minimal clinically important difference of early QW after TKA had not been clarified, a moderate effect size (0.6) was used. Using a significance level of 5% and a power level of 80%, we calculated that a minimum of 45 participants were required for each group.

### Statistical analysis

Statistical analysis was conducted using the IBM SPSS version 26 statistical software package (IBM Corp., Armonk, N.Y., USA). Participants were divided into the LST group and control group. The LST group included participants who performed category 1 sessions (LST and aerobic exercise) more than seven times for three months prior to surgery. The control group included participants who performed less than eight category 1 sessions, category 2 sessions (a general and light exercise) or had no prehabilitation (no exercise) for three months prior to surgery. Propensity score matching was used to balance group characteristics that could affect the LST program’s instructions and formulae. Propensity scores were estimated using a logistic regression model where treatment status was regressed on age, gender, body mass index, and preoperative measurements, including quadriceps strength of the affected leg, knee pain during the QST and TUG, the TUG, the SCT, and JKOM scores. Propensity scores were subsequently used to match participants on a one-to-one basis using the nearest-neighbor method without replacement and a caliper width of 0.2 standard deviations of the logit of the propensity score. Between-group differences in preoperative characteristics and tourniquet time were analyzed before and after matching using standardized mean difference (SMD), with a value exceeding 0.1 indicating a meaningful imbalance.

Measurement changes in both groups and the use of a T-handle cane during the postoperative TUG were then compared using the independent samples t-test for normally distributed continuous data and Mann-Whitney U test for non-normally distributed data. Effect sizes (Cohen’s d) for measurement changes were calculated using the online software available at the following website: https://www.psychometrica.de/effect_size.html.

Furthermore, univariate and multivariable analyses were used to examine the effect of knee swelling, thigh swelling, and Δknee pain on QW and the effect of QW on ΔTUG, respectively. Firstly, correlations between measurement changes were quantified by using Spearman rank correlation coefficients given that all changes were non-normally distributed data except for QW. Thereafter, factors determined to be significantly related to QW and ΔTUG on univariate analysis were entered into multivariable regression models (Enter method) for QW and ΔTUG. To adjust for possible confounding factors, the regression model for QW and ΔTUG included age and tourniquet time (known risk factors for IR injury) [32], and age and gender (known covariates of interest) [1], respectively. Data were assessed for multicollinearity using correlation coefficients and variance inflation factor. A variance inflation factor > 10 indicated multicollinearity.

## Results

**Fig. 1:**
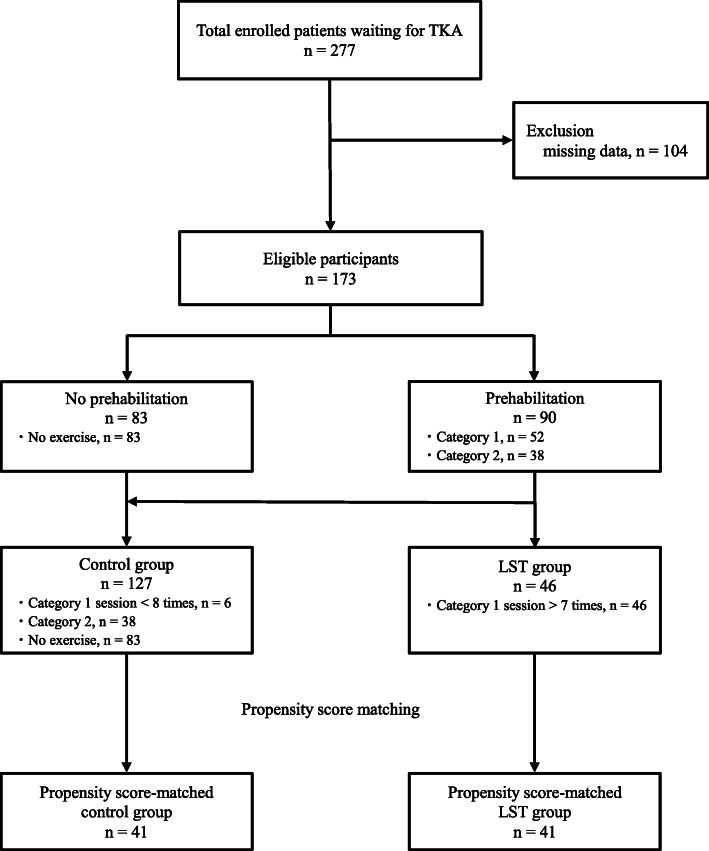
Study flowchart. The LST group included participants who performed category 1 sessions (LST and aerobic exercise) more than seven times for three months prior to surgery. The control group included participants who performed less than eight category 1 sessions, category 2 sessions (a general and light exercise) or had no prehabilitation (no exercise) for three months prior to surgery. Abbreviations: TKA, total knee arthroplasty; LST, low-intensity resistance exercise with slow movement and tonic force generation

During the study period, 277 patients with knee osteoarthritis awaiting unilateral TKA were enrolled. After excluding ineligible participants, 173 eligible participants were ultimately analyzed and divided into the LST (46 participants) and control (127 participants) groups (Fig. 1).

The propensity score model had a c-statistic of 0.76 with a 95% confidence interval of 0.68–0.84, which indicated good discrimination between participants assigned to the LST and control groups. The Hosmer-Lemeshow chi-squared value was 5.67 (degrees of freedom = 8), while the non-significant *p*-value of 0.68 indicated a good model fit. Propensity score matching ultimately selected 41 participants from each group, with Table 1 summarizing the characteristics of all and matched participants. Despite improvements in the covariate imbalance (SMD > 0.1) in gender, prevalence of heart disease, and the TUG (SMD before and after matching: 0.30 and 0.07, respectively) after matching, a small imbalance remained in the prevalence of rheumatoid arthritis, T-handle cane usage, and quadriceps muscle strength of the affected leg (LST group: 1.25 ± 0.09, control group: 1.27 ± 0.15, SMD = 0.18). All imbalanced variables were worse in the LST group than in the control group.

**Table 1:** Preoperative characteristics of participants and tourniquet time in the control and LST groups

	All participants			Matched participants		
	Control (*n* = 127)	LST (*n* = 46)	SMD	Control (*n* = 41)	LST (*n* = 41)	SMD
Age (years), median (IQR)	74 (68, 79)	71 (66, 75)	0.06	71 (66, 75)	71 (67, 75)	0.00
Male, n (%)	40 (31)	4 (9)	0.59	5 (12)	4 (10)	0.08
BMI (kg/m^2^), median (IQR)	26 (23, 28)	25 (23, 28)	0.02	26 (24, 29)	25 (23, 28)	0.00
Current medical history, n (%)						
Heart disease	15 (12)	3 (7)	0.18	2 (5)	3 (7)	0.10
Diabetes	24 (19)	8 (17)	0.04	8 (20)	8 (20)	0.00
Hyperlipidemia	51 (40)	20 (43)	0.07	18 (44)	18 (44)	0.00
Rheumatoid arthritis	1 (1)	2 (4)	0.23	0 (0)	2 (5)	0.32
KL grade 3 of surgical knee, n (%)	14 (12)	5 (11)	0.00	4 (10)	5 (12)	0.08
Contralateral knee, n (%)						
OA and TKA, n (%)	102 (80)	38 (83)	0.06	32 (78)	33 (80)	0.06
Quadriceps strength, median (IQR)	1.3 (1.1, 1.7)	1.4 (1.2, 1.7)	0.05	1.3 (1.1, 1.7)	1.4 (1.2, 1.7)	0.01
T-handle cane usage, n (%)	7 (6)	1 (2)	0.17	0 (0)	1 (2)	0.22
SCT (s), median (IQR)	24 (16, 34)	20 (14, 25)	0.03	21 (17, 31)	22 (16, 27)	0.01
JKOM (points), median (IQR)	37 (25, 49)	32 (25, 45)	0.01	36 (32, 46)	34 (27, 48)	0.00
Tourniquet time (min), median (IQR)	58 (54, 66)	56 (52, 67)	0.01	56, (53, 63)	56 (52, 65)	0.01

Preoperative characteristics and tourniquet time between the groups were compared using standardized mean differences. *Abbreviations*: *LST* low-intensity resistance exercise with slow movement and tonic force generation, *SMD* standardized mean difference, *IQR* interquartile range, *BMI* body mass index, *KL* Kellgren and Lawrence, *OA* osteoarthritis, *TKA* total knee arthroplasty, *SCT* stair climb test, *JKOM* Japanese Knee Osteoarthritis Measure

Table 2 presents the results and measurement changes in both groups. Accordingly, the LST group exhibited significantly lower increase in knee swelling and thigh swelling, QW, and ΔTUG compared to the control group (all, *p* < 0.05), with outcome measures showing a medium to large effect size difference. Moreover, no significant differences in Δknee pain during the QST and TUG and T-handle cane usage during the TUG were observed between both groups (all, *p* > 0.05), with outcome measures showing a small effect size difference.

**Table 2:** Results of pre- and postoperative measurements and changes therein among control and LST groups

Parameters	Control (n = 41)			LST (n = 41)			Change	
	Pre	Post	Change	Pre	Post	Change	p	ES
Knee circumference (1 cm) (cm)	39 (37, 41)	43 (40, 45)	10 (8, 12)	40 (37, 41)	43 (41, 44)	9 (7, 10)	0.00	0.65
Knee circumference (10 cm) (cm)	44 (41, 47)	47 (44, 50)	9 (4, 10)	44 (41, 46)	46 (44, 48)	6 (3, 8)	0.01	0.57
Thigh volume (× 10^2^ cm^3^)	13 (11, 14)	15 (13, 16)	21 (13, 23)	12 (11, 13)	14 (13, 15)	13 (12, 18)	0.00	0.67
Knee pain during the QST (mm)	10 (0, 29)	68 (30, 80)	40 (20, 68)	7 (0, 27)	62 (41, 76)	44 (17, 65)	0.72	0.08
Knee pain during the TUG (mm)	20 (8, 40)	30 (27, 60)	20 (−10, 39)	18 (5, 43)	53 (43, 70)	32 (2, 48)	0.16	0.32
Quadriceps strength (Nm/kg)	1.2 (1.0, 1.5)	0.4 (0.3, 0.5)	− 69 (−79, −59)	1.3 (1.1, 1.4)	0.5 (0.4, 0,7)	−57 (−63, −46)	0.00	0.84
TUG (s)	7 (7, 8)	18 (14, 22)	137 (86, 191)	7 (6, 8)	14 (12, 18)	97 (68, 132)	0.00	0.68

*Note*. Results of pre- and postoperative measurements and changes therein are presented as median (IQR). *p* values are those for independent samples t-test or Mann-Whitney U test. ES indicates the effect size (Cohen’s d) quantifying difference in measurement changes between both groups

*Abbreviation*: *LST* low-intensity resistance exercise with slow movement and tonic force generation, *ES* effect size, *TUG* timed up and go test

Correlations between measurement changes and their significance in all participants are detailed in Table 3. Univariate analysis in all participants showed that QW was significantly related to knee swelling (1 cm), thigh swelling, and Δknee pain during the QST (all, *p* < 0.05), while ΔTUG was significantly related to knee swelling (1 cm), thigh swelling, and QW (all, p < 0.05). Although not shown in Table 3, knee swelling (1 cm) was found to be significantly associated with knee swelling (10 cm) (r = 0.58; *p* = 0.00). Results of the multivariable regression analyses are shown in Tables 4 and 5. Considering multicollinearity between knee swelling (1 cm) and thigh swelling, we examined multivariable regression equations with each outcome as the independent variable (Table 4: knee swelling [1 cm]; Table 5: thigh swelling). Multivariable regression analyses identified factors independently associated with QW and ΔTUG in all participants (ANOVA all *p* = 0.00), with a relatively small explained variance in dependent variables (all R^2^ = 0.10). Even after adjusting for possible confounders, QW was found to be significantly associated with knee swelling (1 cm), thigh swelling, and Δknee pain during the QST, while ΔTUG was determined to be significantly associated with QW. Multicollinearity was not detected in any of the multivariable regression models (variance inflation factor < 2).

**Table 3:** Correlations between measurement changes and their significance in all participants

Variables	Knee swelling (1 cm) (%)	Thigh swelling (%)	ΔKnee pain during the QST (mm)	QW (%)	ΔKnee pain during the TUG (mm)
	r (p)	r (p)	r (p)	r (p)	r (p)
Thigh swelling (%)	0.84 (0.00)	–	–	–	–
ΔKnee pain during the QST (mm)	0.15 (0.05)	0.19 (0.01)	–	–	–
QW (%)	−0.27 (0.00)	−0.26 (0.00)	−0.23 (0.00)	–	–
ΔKnee pain during the TUG (mm)	0.10 (0.19)	0.08 (0.32)	0.30 (0.00)	−0.10 (0.17)	–
ΔTUG (%)	0.23 (0.00)	0.23 (0.00)	0.01 (0.93)	−0.40 (0.00)	0.02 (0.84)

*Note*. *N* = 173, male = 44. r stands for Spearman correlation coefficients. The Δ symbol indicates pre- and postoperative measurement changes

*Abbreviations*: *QW* quadriceps weakness, *QST* quadriceps strength test, *TUG* timed up and go test

**Table 4:** Factors independently influencing changes in quadriceps strength and TUG in all participants (independent variable: knee swelling [1 cm])

Dependent variables	Independent variables	Standardized β coefficient	p
QW	Age (year)	0.06	0.44
	Tourniquet time (min)	− 0.05	0.47
	Knee swelling (1 cm) (%)	−0.23	0.00
	ΔKnee pain during the QST (mm)	−0.17	0.03
ΔTUG	Age (year)	−0.04	0.60
	Gender	−0.07	0.39
	Knee swelling (1 cm) (%)	−0.05	0.55
	QW (%)	−0.29	0.00

*Note*. N = 173, male = 44. Standardized β indicates the adjusted regression coefficient. The Δ symbol indicates pre- and postoperative measurement changes

*Abbreviations*: *QW* quadriceps weakness, *QST* quadriceps strength test, *TUG* timed up and go test

**Table 5:** Factors independently influencing changes in quadriceps strength and TUG in all participants (independent variable: thigh swelling)

Dependent variables	Independent variables	Standardized β coefficient	p
QW	Age (year)	0.08	0.31
	Tourniquet time (min)	−0.04	0.56
	Thigh swelling (%)	−0.24	0.00
	ΔKnee pain during the QST (mm)	−0.16	0.04
ΔTUG	Age (year)	−0.03	0.64
	Gender	−0.07	0.36
	Thigh swelling (%)	−0.06	0.45
	QW (%)	−0.30	0.00

*Note*. N = 173, male = 44. Standardized β indicates the adjusted regression coefficient. The Δ symbol indicates pre- and postoperative measurement changes

*Abbreviations*: *QW* quadriceps weakness, *QST* quadriceps strength test, *TUG* timed up and go test

## Discussion

This study showed that the LST group exhibited superior and more clinically relevant results in terms of increase in knee and thigh swelling, QW, and walking disability immediately after TKA compared to the control group. However, no significant differences in Δknee pain during the QST and TUG had been observed between both groups.

Some studies utilizing human models have shown that QW can be associated with knee swelling, knee pain, and quadriceps muscle atrophy, which are partially caused by tourniquet use during TKA [12–14]. Thus, stronger resistance to tourniquet-induced oxidative stress and inflammatory response may be assumed to suppress knee swelling, knee pain, and quadriceps muscle loss, resulting in lower QW and walking disability immediately after TKA. IPC and EPC have been demonstrated to attenuate IR-induced oxidative stress and inflammatory responses in various organs by conferring hypoxic and exercise stimulation to therapeutic organs before exposing them to subsequent lethal IR injury [17–20]. Hence, this study focused on a form of exercise called LST that confers both hypoxic and exercise stimulation to working skeletal muscle and subsequently investigated whether preoperative LST program would suppress knee and thigh swelling and knee pain, resulting in decreased QW and walking disability immediately after TKA.

Several animal studies have revealed that IPC and EPC exert beneficial effects on edema formation in various organs by reducing IR-induced oxidative stress and inflammatory responses [33, 34]. Similarly, our data showed that the LST group exhibited significantly lower knee (1 and 10 cm) and thigh swelling immediately after TKA compared to the control group, indicating that LST could effectively attenuate tourniquet-induced edema formation in the muscles of the affected limb. However, the knee swelling (1 cm) observed herein may reflect not only the extent of muscle edema around the knee joint but also intra-articular blood accumulation considering that knee swelling (1 cm) can be quantified through changes in the knee circumference measured at 1 cm proximal to the upper edge of the patella. Blood accumulation immediately after TKA can be associated with postoperative blood loss and drainage volume (intra-articular blood accumulation = postoperative blood loss − drainage volume). An earlier study described that postoperative blood loss was associated with sex and tourniquet time [35]. The present study, however, found no significant difference in sex and tourniquet time between both groups, thereby suggesting nearly similar postoperative blood loss in both groups. Given no large difference in postoperative blood loss between both groups, as well as the use of the same drainage method in all participants, blood accumulation immediately after TKA can be considered similar in both groups. Furthermore, univariate analysis including all participants showed a significant positive association between knee swelling (1 cm) and knee swelling (10 cm), suggesting that knee swelling (1 cm) can reflect muscle edema around the knee joint. Based on the aforementioned findings, the significant difference in knee swelling (1 cm) between both groups can be reasonably attributed to IR-induced muscle edema around the knee joint.

Our data showed no significant difference in Δknee pain during the QST and TUG between both groups immediately after TKA. Studies have shown that the increase in interleukin-1 beta (IL-1β) in IR-affected muscle can be associated with not only edema formation but also ischemic myalgia, characterized by local mechanical hypersensitivity, decreased muscle strength, and decreased voluntary activity [36, 37]. The present study showed that the LST group had lower inflammation-induced knee and thigh swelling than the control group, suggesting that the LST group had lower IL-1β expression. Thus, we can conjecture that LST suppresses the increase in IL-1β-induced mechanical hypersensitivity in the IR-affected quadriceps muscle, as well as Δknee pain during the QST and TUG. However, our data showed no significant difference in Δknee pain between both groups perhaps due to unadjusted factors associated with knee pain immediately after TKA, including psychosocial variables [38] and heterogeneity in the amount of prescribed medicine or rescue analgesics for postoperative pain management. Taken together, the aforementioned factors likely contributed to knee pain during the QST and TUG, leading to no significant difference between both groups.

The current study found that the LST group had significantly lower early QW after TKA compared to the control group. Moreover, multivariable analysis including all participants revealed that QW was significantly associated with knee swelling (1 cm), thigh swelling, and Δknee pain during the QST. Given no significant difference in Δknee pain during the QST between both groups, the difference in early QW after TKA could be mainly attributed to knee swelling (1 cm) and thigh swelling. As mentioned previously, the significant difference in knee swelling (1 cm) between both groups might be mainly attributed to muscle edema around the knee joint, which could have partly contributed to QW. Several studies have demonstrated that intra-articular knee injection of fluid can raise intra-articular pressure and consequently increase the discharge of group II afferents from the knee, facilitating Ib inhibition of the quadriceps motoneuron pool [39, 40]. During the QST, muscle edema around the knee joint might have contributed to increased intra-articular pressure, resulting in inhibited quadriceps muscle activation. Additionally, studies have revealed that tourniquet use may increase susceptibility to quadriceps muscle atrophy, characterized by activation of cell death and catabolic processes after TKA [13, 14]. Given that thigh swelling can be attributed to IR injury, it may indicate the extent of IR injury and IR-induced quadriceps muscle atrophy. As discussed so far, the LST group had lower quadriceps muscle inactivation and atrophy compared to the control group, partly accounting for the significant difference in early QW between the groups.

Our results also found that the LST group had significantly lower ΔTUG immediately after TKA compared to the control group. Moreover, multivariable analysis that included all participants showed that ΔTUG was significantly associated with QW. Given that quadriceps muscle strength has been known to affect walking capacity among patients with knee osteoarthritis and those who had undergone TKA [41, 42], we considered that QW had affected ΔTUG. Furthermore, the current study showed that the LST group had significantly lower early QW after TKA compared to the control group. Thus, the LST group had lower ΔTUG immediately after TKA compared to the control group, suggesting that addressing early QW is imperative in reducing walking disability immediately after TKA.

### Study limitations

There are several limitations that need to be considered. First, the study included a small number of each group participants. Second, this was a single-center retrospective study; accounting for all unmeasured or unknown confounders affecting the outcomes was impossible, even after propensity score matching. Third, some variables remained imbalanced after propensity score matching. However, it is important to note that most imbalanced variables were worse in the LST group than that in the control group, suggesting that preoperative LST program may have improved early QW even in cases with relatively low physical function. Finally, given that QW was assessed only on postoperative day 4, it remains uncertain whether early QW suppression can optimize long-term postoperative recovery. In future, a large-scale multicenter randomized controlled trial with long-term follow up is needed to address these limitations.

## Conclusions

The present study showed that preoperative LST program exerted beneficial effects on knee and thigh swelling, QW, and walking disability immediately after TKA. Future research addressing the limitations of this study is nonetheless needed to confirm the validity of our findings.

## Data Availability

The datasets used and/or analyzed during the current study are available from the corresponding author on reasonable request.

## Abbreviations

TKA: Total knee arthroplasty
QW: Quadriceps weakness
IR: Ischemia-reperfusion
IPC: Ischemic preconditioning
EPC: Exercise preconditioning
LST: Low-intensity resistance exercise with slow movement and tonic force generation
QST: Quadriceps strength test
TUG: Timed up and go test
SCT: Stair climb test
JKOM: Japanese Knee Osteoarthritis Measure
VAS: Visual analog scale
SMD: Standardized mean difference
